# The Adamantaneland Revisited

**DOI:** 10.1021/acs.jpca.5c06236

**Published:** 2025-11-04

**Authors:** Pedro H. Antunes Silva, Amir L. Perlin, Cleverson J. F. de Oliveira, Ricardo R. Oliveira, Pierre M. Esteves

**Affiliations:** † Instituto de Química, Universidade Federal do Rio de Janeiro, Av. Athos da Silveira Ramos, 149, CT A-622, Cid. Univ., Rio de Janeiro, RJ 21941-909, Brazil; ‡ Centro de Pesquisa e Desenvolvimento Leopoldo Miguez de Mello, CENPES (Petrobras), Av. Horácio de Macedo, 950 - Ilha do Fundão, Rio de Janeiro RJ 21941-598, Brazil

## Abstract

Diamondoids are a class of rigid, cage-like hydrocarbons found exclusively in petroleum on Earth, renowned for their exceptional thermal and thermodynamic stability. Their resistance to decomposition under geological conditions makes them valuable as geological markers. However, a limited understanding of the processes leading to their formation has hindered their broader application, particularly in comparison to conventional biomarkers. This study explores the formation pathways of the simplest diamondoid, adamantane, via a carbocationic mechanism originating from isomeric hydrocarbons. The thermodynamic stability of adamantane and the 1-adamantyl cation was assessed relative to their isomers using the M06–2X/cc-pVTZ level of theory. The results confirm that adamantane is the most stable C_10_H_16_ isomer; however, its corresponding carbocation, 1-adamantyl, is not the most stable C_10_H_15_^+^ species. Instead, the most stable carbocations are allylic species containing the 1-methylhexahydroindene ring system, which is associated with the naphthenic fraction of petroleum. These findings suggest that hydrocarbons containing the 1-methylhexahydroindene ring system may have a geochemically relevant connection to diamondoid formation, and their ratios can be of geochemical interest.

## Graphical abstract

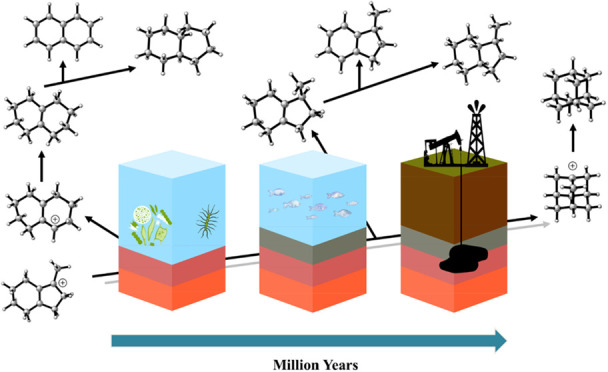

## Introduction

Diamondoids are part of a class of hydrocarbons found in petroleum, characterized by their rigid cage-like structure and high thermal and thermodynamic stability. Due to their resistance to environmental conditions, these compounds have become valuable for assessing petroleum degradation and biodegradation levels, establishing oil–oil correlations, basin characterization, and evaluating the extent of petroleum cracking undergone by specific oil.^1^ Alongside biomarkers, this class of molecules provides essential information about a given petroleum reservoir, granting these compounds unique applications in the oil and gas industry.^2^

Diamondoids were first isolated in 1933 from petroleum sources collected in Czechoslovakia^3^ and occur in the saturated hydrocarbon fraction of petroleum. Schleyer reported the synthesis of the simplest diamondoid, adamantane^4^ in 1957, while the synthesis of larger diamondoids such as diamantane (1965),^5^ triamantane (1966),^6^ and antitetramantane^7^–along with other larger species, were reported later (Figure 1).

**Figure 1:**
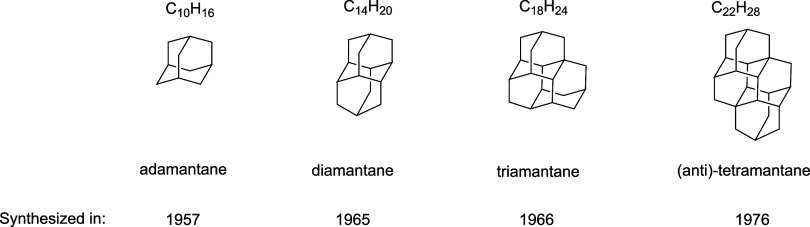
Some representative diamondoids already synthesized.

Regarding the processes involved in the formation of diamondoids, it is recognized by the geochemical community that they are generated through the exposure of paraffin and cycloparaffin fractions to thermal cracking above 350 °C, in the presence of acidic conditions during the oil formation window.^8^ Diamondoids do not provide sufficient information to uncover the entire geological history of petroleum, as the exact events leading to the formation of these hydrocarbons remain unknown. Thus, it is only acknowledged that, like most compounds in petroleum, diamondoids are formed through a variety of chemical reactions of kerogen, which may involve the defunctionalization of this raw material, rearrangements, cleavage of C–C bonds, condensation, as well as oxidation and reduction reactions.^9^

von R. Schleyer and col. show that adamantane could be obtained through the isomerization mediated by the rearrangement of carbocations from endo and exotrimethylenenorbornane under superacid conditions.^4^ Under these conditions, carbocations are formed and undergo isomerization through Wagner–Merweein (*aka* 1,2-sigmatropic) rearrangements, affording the carbocations that are precursors of adamantane.^10^ Adamantane was later recognized as the most stable C_10_H_16_ isomer, and because of that, when subjected to superacid conditions, all C_10_H_16_ isomers would convert to adamantane through carbocation rearrangement, followed by a hydride transfer.^11^ The highly symmetrical 1-adamantyl cation is rigid and relatively stable due to hyperconjugation effects (Figure 2). In SbF_5_, for instance, 1-fluoroadamantane is ionized to form the famous bridged 1-adamantyl cation, which has ^1^H NMR signals at 5.40, 4.52, and 2.67 ppm^12^ assigned to the hydrogen atoms bonded at the γ, β, and δ positions (Figure 2).

**Figure 2:**
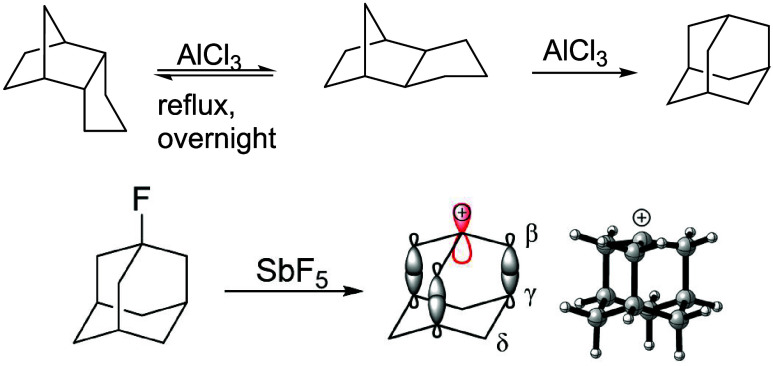
Synthesis of adamantane (top) and ionization of 1-fluoro-adamantane in SbF_5_ to obtain the 1-adamantyl cation (bottom).

Notably, the γ hydrogens are attributed to the most deshielded signals, even though the CH_2_–β groups are closer to the positively charged center.^12^ The reason for the unusual observation was attributed to the so-called “cage effect”, where the virtual p orbital of the carbocation interacts with the bridged C–H bonds, causing the deshielding of these protons.^12^ Olah proposed that the remarkable stability of the 1-adamantyl cation is due to hyperconjugation phenomena involving lateral σ bonds and the virtual p orbital, resulting in a σ–π* hyperconjugation effect that not only explains the relatively high stability of this carbocation but also the deshielding of the γ protons.^13^ A similar effect is indicated by ^13^C NMR of 1-adamantyl cation, which shows a chemical shift of 300 ppm for the bridgehead carbon, 65.7 ppm for the C_β_, and 86.8 ppm for the C_γ_ ^14^ supporting the “cage effect” or CC hyperconjugation with the vacant π orbital.^15^

These special positive charge stabilization effects of the 1-adamantyl cation are responsible for explaining its formation as resulting from carbocation isomerization reactions in a superacid medium. Engler, Farcasiu, and Schleyer^16^ investigated the process involved in the laboratory formation of adamantane, seeking to uncover the possible intermediate carbocations involved in its formation. In their work, the authors proposed rearrangement pathways involving more than a thousand intermediate species in the formation of adamantane, which they called “adamantaneland”.^16^ These authors investigated the stability of the intermediates that were supposed to be involved in the rearrangement route of carbocations for the formation of adamantane using molecular mechanics methods and experimental data on the stability of certain known carbocations (Figure 3).^16^

**Figure 3:**
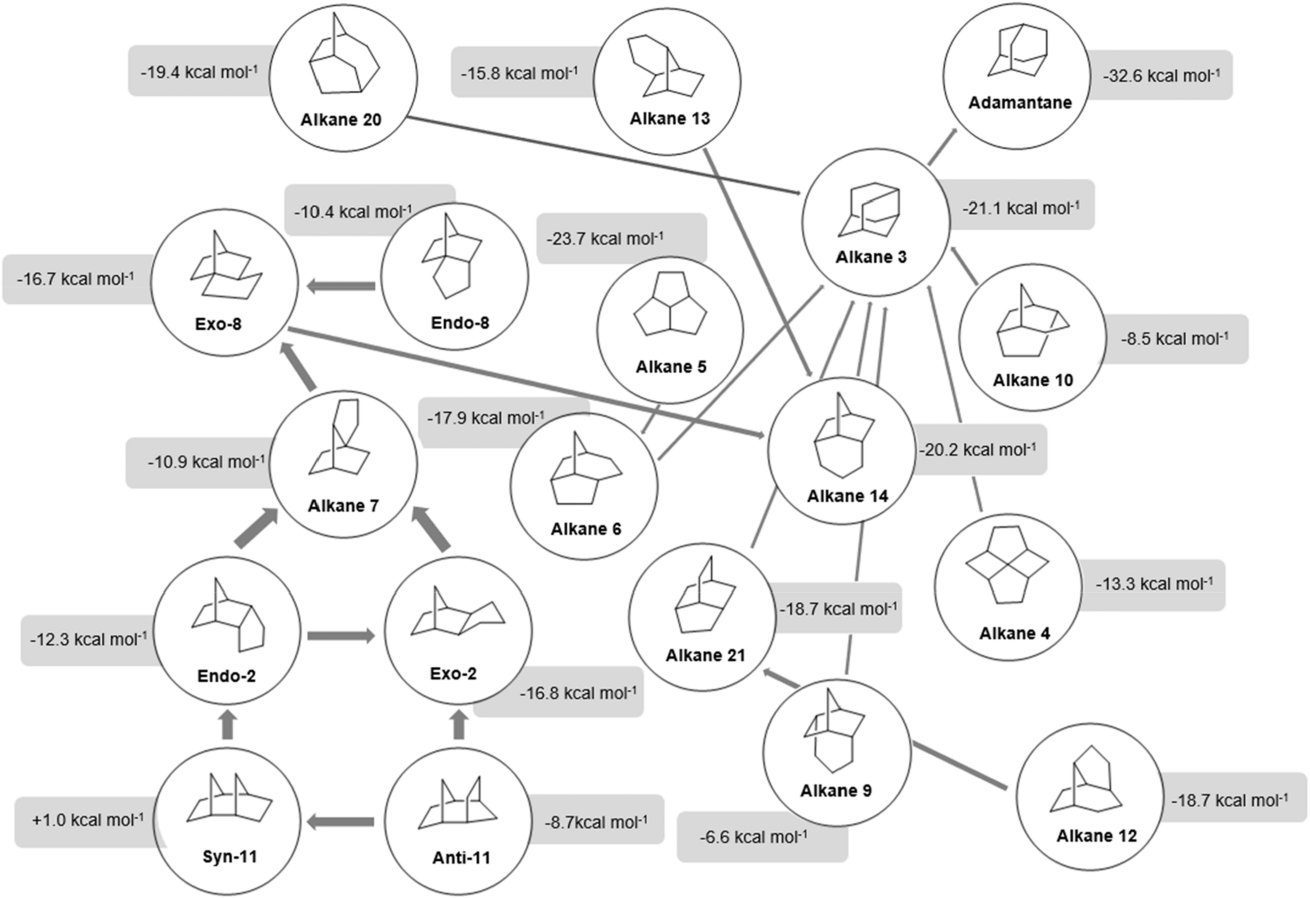
Intermediates involved in adamantane formation, according to Engler and collaborators.

Curiously, adamantane (C_10_H_16_) is isomeric to the monoterpenes (C_10_H_16_), a well-known class of natural products, whose formation process is well understood and occurs through the rearrangement of intermediate carbocations inside enzymes in various living organisms.^17^ Monoterpenes^18^ are among the most common plant secondary metabolites, especially prevalent in essential oils of aromatic plants like mint, citrus, pine, and eucalyptus. Structurally, they are composed of two isoprene units (C_10_H_16_), and due to their volatility and lipophilicity make them ideal for ecological roles^19^ such as chemical communication (*e.g.*, attracting pollinators or deterring herbivores), sexual pheromones, etc. Monoterpenes are formed from a common precursor carbocation, the linalyl cation, which, after cyclization, undergo a series of rearrangements and hydride and alkyl group migrations, affording the various monoterpenes found in nature.^17^ Similarly, one may think that larger terpenes, such as biomarkers, may also ionize to the corresponding carbenium ions when exposed to acid sites and/or relatively high temperatures within the source rock and produce some C_10_H_15_^+^ or higher carbocations. These carbocations then might evolve through rearrangement processes toward thermodynamic sinks, such as the 1-adamantyl cation and related species, which will eventually afford the diamondoid family.

With this hypothesis in mind, this work revisits the study by Engler, Farcasiu, and Schleyer on the process involved in the formation of adamantane, employing more modern quantum chemical methods, aiming to verify a possible pathway of the formation of the diamondoid family starting from some point of the cationic terpene cascade. Additionally, an analysis of the thermodynamic stability of the known C_10_H_16_ hydrocarbons cataloged on the National Institute of Standards and Technology (NIST) platform was conducted to determine whether adamantane could indeed be the most stable C_10_H_16_ isomer^11^ will be presented, as well as the thermodynamic stability of several C_10_H_15_^+^ carbocations to evaluate their energies relative to the 1-adamantyl cation.

## Computational Details

Aiming to evaluate the stability of the C_10_H_16_ isomers, a search of compounds with such a molecular formula was conducted on the National Institute of Standards and Technology (NIST) platform,^20^ resulting in 242 compounds found. In the initial screening, 22 compounds on the platform had issues with their descriptions (not being isomers, lacking molecular formulas, names, CAS numbers, or any other type of identifier) and were therefore excluded from the study. Next, the thermodynamic energetics of all these isomers were computed and compared to adamantane. All optimization and frequency calculations of the structures were performed using the Gaussian16 software using the M06–2X functional using the 6–31G­(d,p) basis set (initial screening) and then reoptimized with the cc-pVTZ basis sets. The choice of the functional was based on literature data that indicate M06–2X as one of the most used functionals in the study of carbocations due to its low computational cost and good agreement with experimental data.^21^

The AUTOMATON software^22^ was used to search for the low lying energy carbocation isomers with the formula C_10_H_15_^+^, with calculations performed at the DFT level with PBE functional in the 6–31G­(d) basis set, with an initial population of 5N (where N is the number of atoms that exist on the system) resulting in a total of 125 different structures. After the calculations converged, the geometries and vibrational frequencies of the 10 lowest-energy isomers were optimized at the M06–2X/cc-pVTZ level. The energy of these species was compared with the energy of the 1-adamantyl cation, presumed to be the most stable C_10_H_15_^+^ cation.

## Results and Discussion

Initially, the thermodynamic stability data of the intermediates proposed by Engler et al.,^16^ evaluated in the original work employing molecular mechanics calculations, were reevaluated, using more modern DFT chemistry methods. According to the original work, adamantane would be the neutral species of lowest energy, with a heat of formation of −32.6 kcal mol^–1^. Based on the idea that adamantane would be the most stable isomer, the energy of all proposed species was compared to this hydrocarbon (Figure 4). The relative energies of these isomers were calculated at the M06–2X/cc-pVTZ level to reassess the stability of these species.

**Figure 4:**
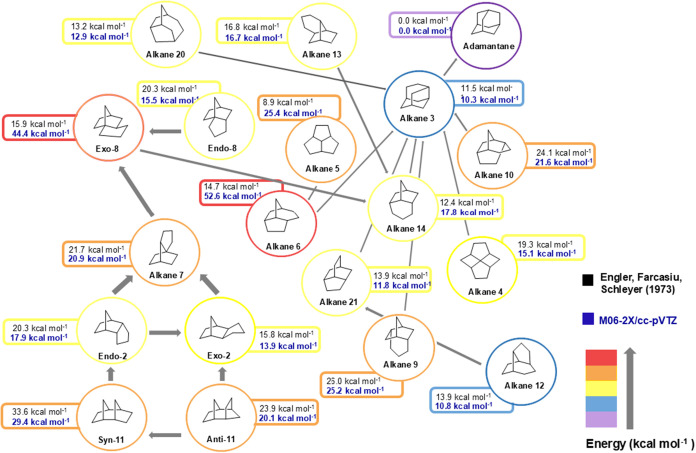
Comparison of the free energy of isomers obtained by Engler and collaborators^16^ and those determined using M06–2X/cc-pVTZ.

Comparing the data obtained, it was verified that only two of the evaluated isomers, the alkane **6** and **Exo-8**, showed a significant deviation when compared to the original work. This difference in energy can be understood in terms of the molecule’s stereochemistry, such that the various eclipsed or partially eclipsed bonds and the existing strained rings could be responsible for the observed energy elevation of this hydrocarbon (Figure 5). Alkane **5** also showed a significant energy variation relative to adamantane, which was not present in the original work. This also seems to be related to the steric tensions present in the molecule.

**Figure 5:**
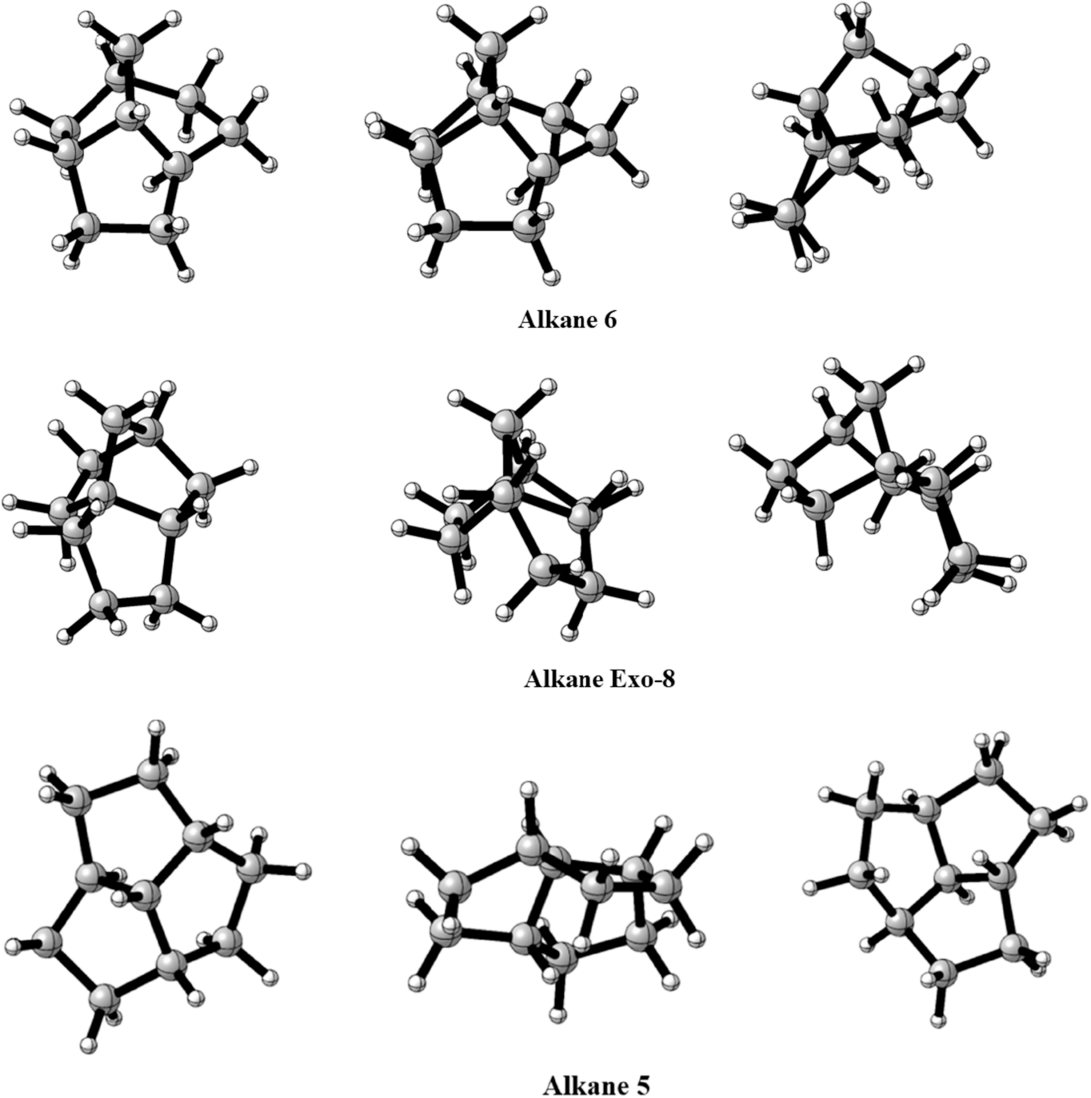
Structure of alkanes exo-8, 5, and 6, optimized at M06–2X/cc-pVTZ level.

In addition to these cases, no other species showed such a significant variation in energy, with more detailed structural information about them available in the Supporting Information section of this work. Thus, it was possible to verify that, although some discrepancies were observed using modern DFT methods, the energy of the hydrocarbons isomeric to adamantane did not present major changes. Thus, the main picture of the original work remains valid, with adamantane remaining the most stable hydrocarbon. Therefore, an important aspect to be investigated is whether one could state that adamantane is the most stable C_10_H_16_ hydrocarbon. By applying the M06–2X/6–31G­(d,p) level of theory, the thermodynamic stability of those 220 isomers of adamantane listed in the NIST database was evaluated, and it was not possible to identify any species with a relative Δ*G* to adamantane lower than 10 kcal mol^–1^. Only the isomer of adamantane, the 2,5-methane-1*H*,indene,octane, showed energy close to it, with a relative Δ*G* of 10.4 kcal mol^–1^, followed by perhydrotriquinacene (Δ*G* = 11.4 kcal mol^–1^). Meanwhile, monoterpene isomers, such as limonene, camphene, and γ-terpinene, exhibit a free energy variation of +28.6, +26.2, and +25.2 kcal mol^–1^, respectively, relative to adamantane (Figure 6).

**Figure 6:**
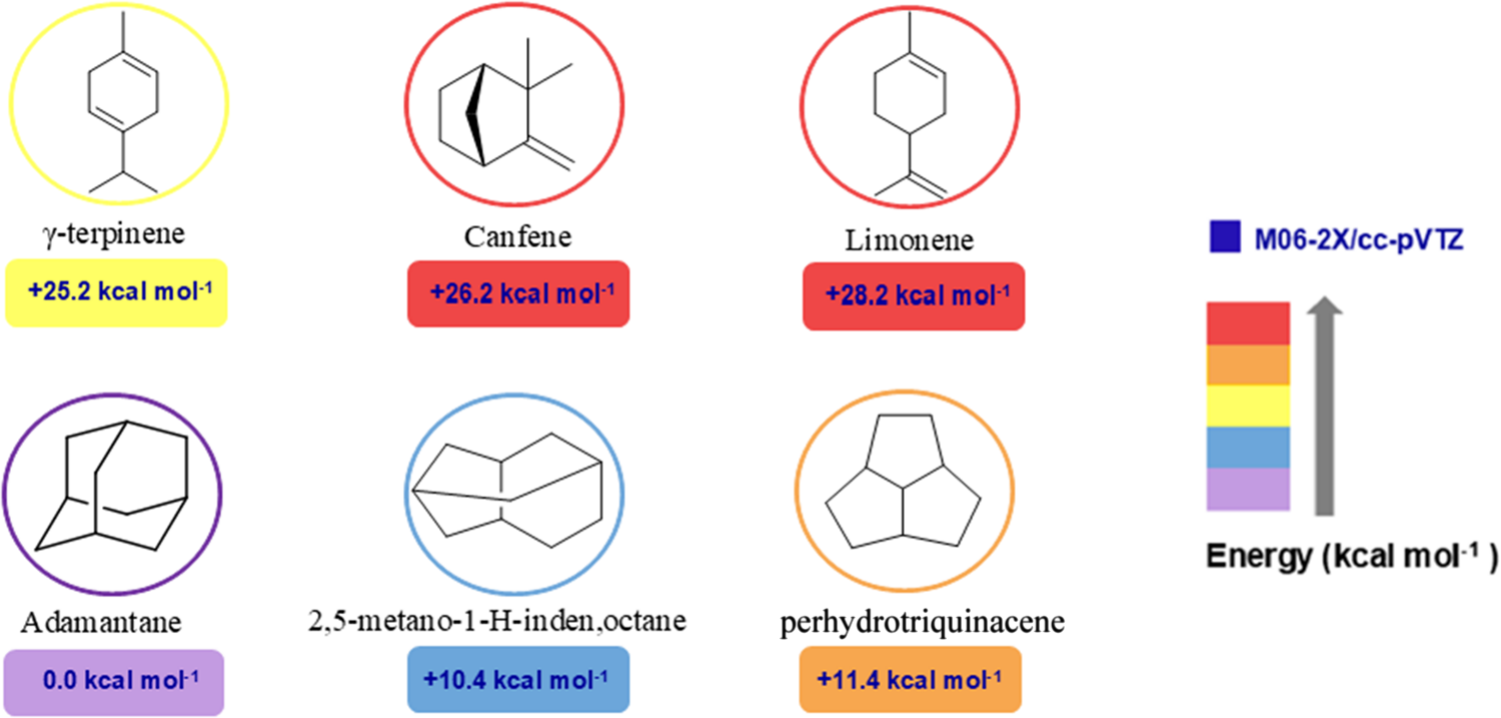
Structure of the lowest energy isomers relative to adamantane and their natural product isomers (Δ*G* predicted at M06–2X/cc-pVTZ level).

These results support the initial hypothesis and Olah’s statement that adamantane would be the most stable known C_10_H_16_ hydrocarbon, suggesting the processes involved in its formation could indeed occur through carbocation rearrangement. However, to confirm this, it is necessary to evaluate the thermodynamic stability of the 1-adamantyl cation compared to its C_10_H_15_^+^ carbocations isomers. Initially, the carbocations proposed by Engler and co-workers as intermediate species involved in the formation of adamantane were revised, and the theoretical values obtained were compared with the original data. It is noteworthy that, according to them, the energy values obtained are relative to the *tert*-butyl cation, which has an experimental Δ*H*_f_^0^ of 170.4 kcal mol^–1^, corrected according to the degree of branching of the molecule−β branching 3 kcal mol^–1^ for secondary cation and 1.5 kcal mol^–1^ for tertiary ones, in addition to 12 kcal mol^–1^ for higher electronic stability of the tertiary carbocation compared to the secondary one, and for bridgehead norbornyl-type cations, 5 kcal mol^–1^.^16^

Taking the 2-adamantyl cation as a reference, the energy of the intermediate species presented by the authors was calculated and compared with the data obtained using modern theoretical chemistry methods, as illustrated below (Figure 7).

**Figure 7:**
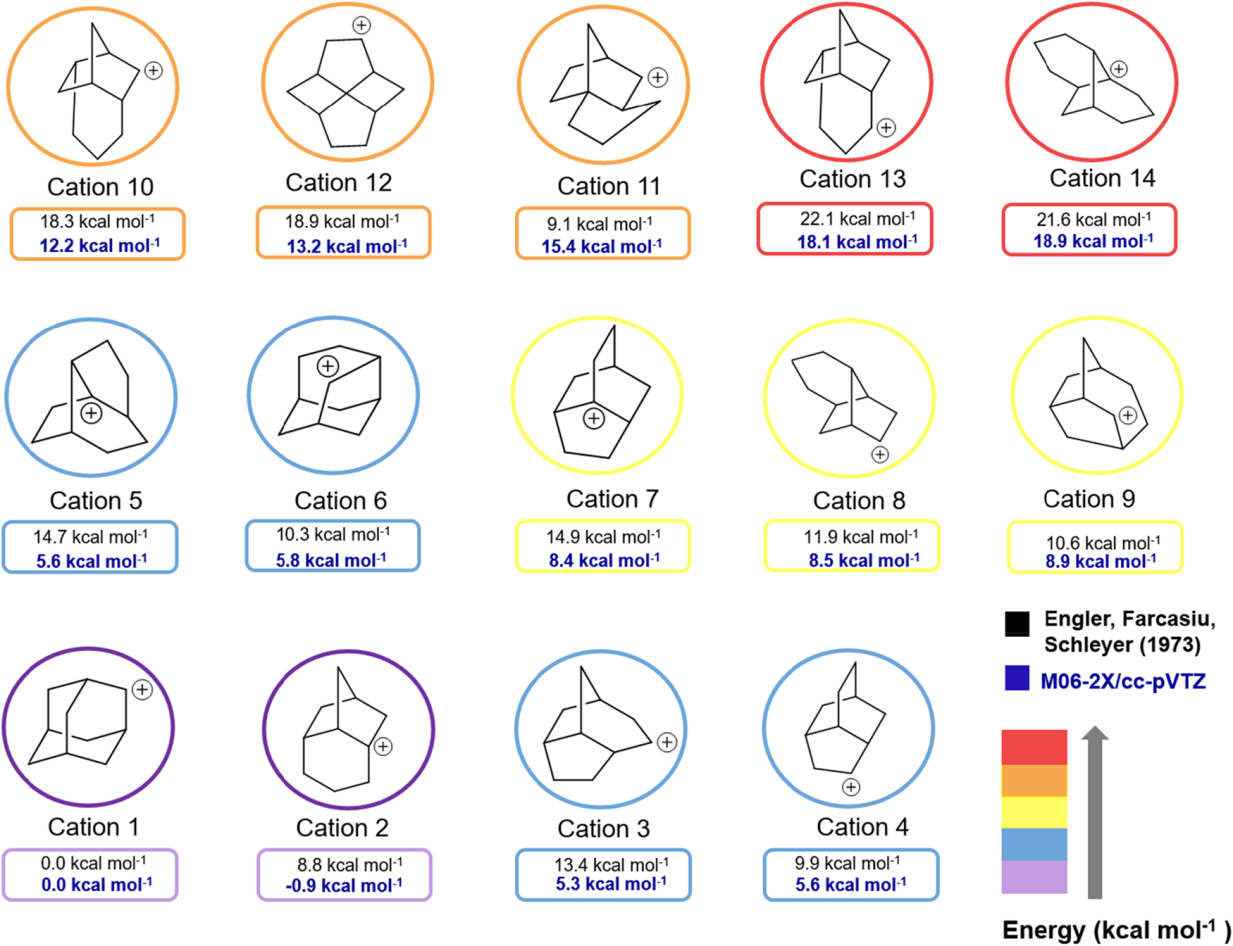
Comparison between the energies of isomeric carbocations presented by Engler et al. and with values computed at M06–2X/ccVTZ.

The calculations indicate that carbocation **2** is more stable than 2-adamantyl cation by 0.9 kcal mol^–1^. Note that the carbocation **2** skeleton is related to the norbornyl cation. It is important to highlight that 1 kcal mol^–1^ represents the threshold of chemical accuracy, which can generally only be achieved at the coupled cluster level with very large basis sets, typically of quintuple-zeta quality. Therefore, both cations (1 and 2) are candidates for the global minimum (Figure 8).

**Figure 8:**
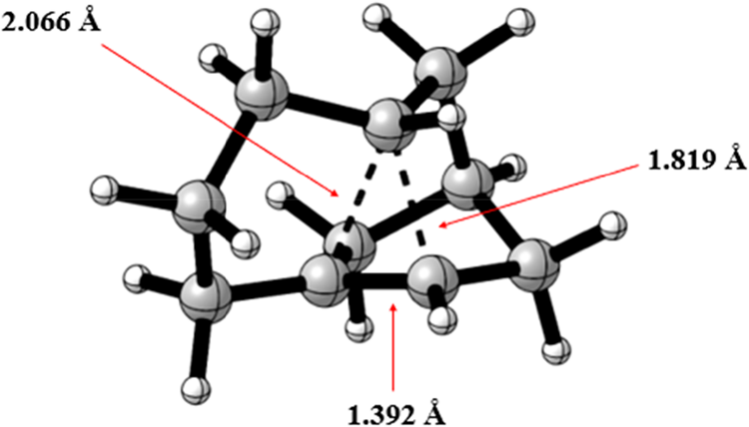
Optimized structure of carbocation 2.

The most interesting aspect of this carbocation is the stabilization of the positive charge, favored by the 3-center-2-electrons (3c2e) system, which can only be formed thanks to the presence of the norbornyl system in the molecule. Supported by the existence of a bicyclic system, the main characteristic factor of these nonclassical carbocations is the bond length values, with the basal bonds measuring 1.39 Å and the bridge bonds measuring 1.82 and 2.07 Å (Figure 9)–values that align with the experimental data for the symmetrical norbornyl cation obtained by XRD.^23^

**Figure 9:**
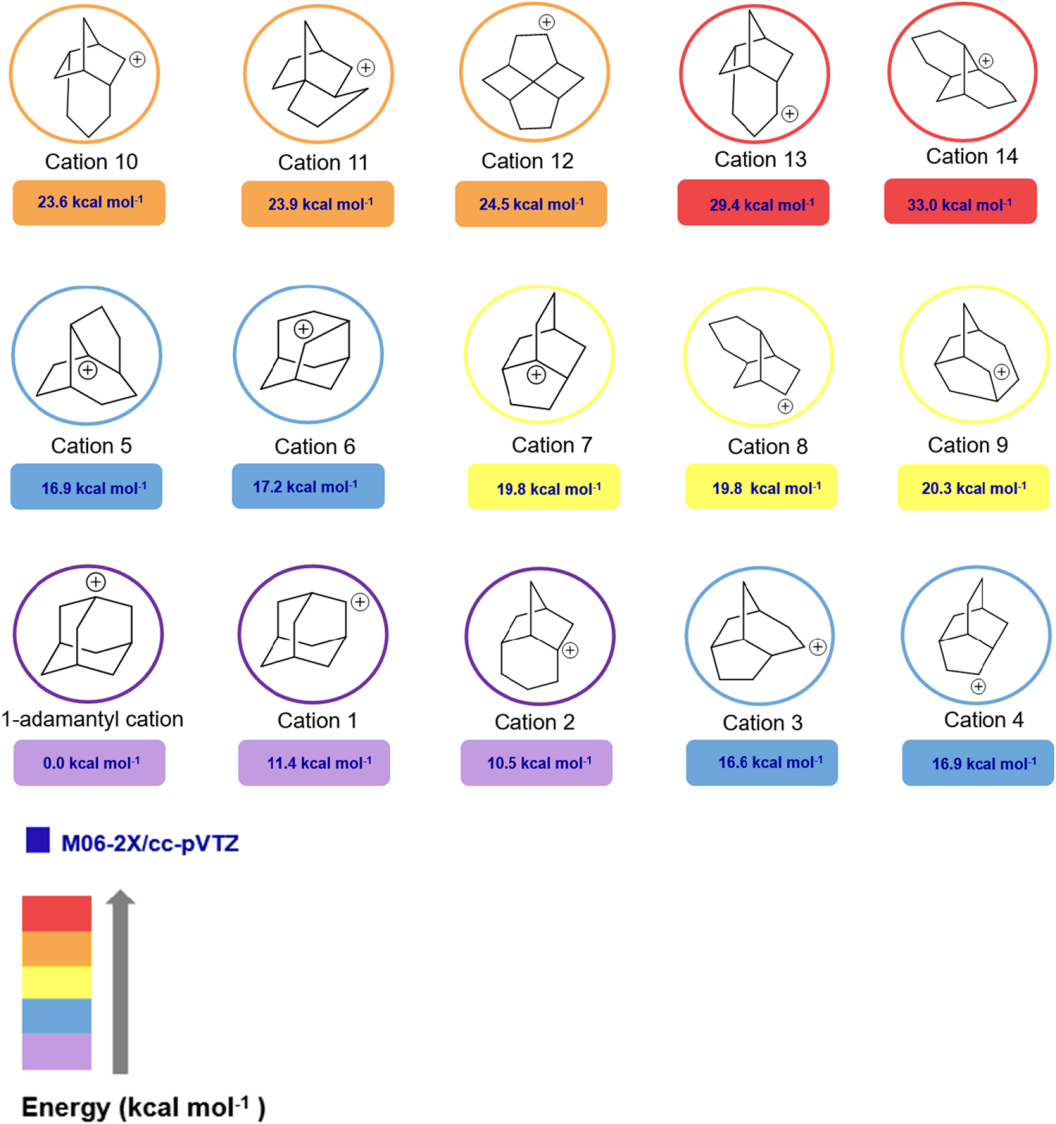
Comparison of the energy of isomeric carbocations with 1-adamantyl cation.

Noteworthy, none of the above-mentioned carbocations are more stable than the 1-adamantyl cation (Figure 10).

**Figure 10:**
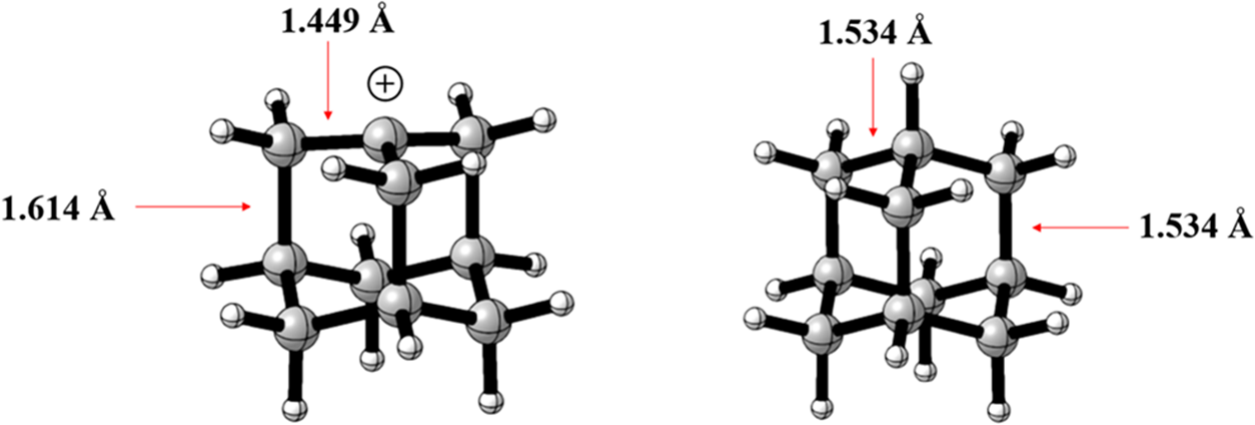
Calculated structure of 1-adamantyl cation by M06–2X/cc-pVTZ.

Since the number of isomeric structures is higher than the ones reported by Engler et al., the AUTOMATON software^16^ was used to search for isomeric structures of the 1-adamantyl cation. This program is based on a global minima search using genetic algorithms.^22^ The list generated by the algorithm of the isomeric cations of formula C_10_H_15_^+^ shows that the 1-methyl-2,3,4,5,6,7-hexahydro-1*H*-indene-1-yl cation (Figure 11) is 12.7 kcal mol^–1^ more stable than the 1-adamantyl cation.

**Figure 11:**
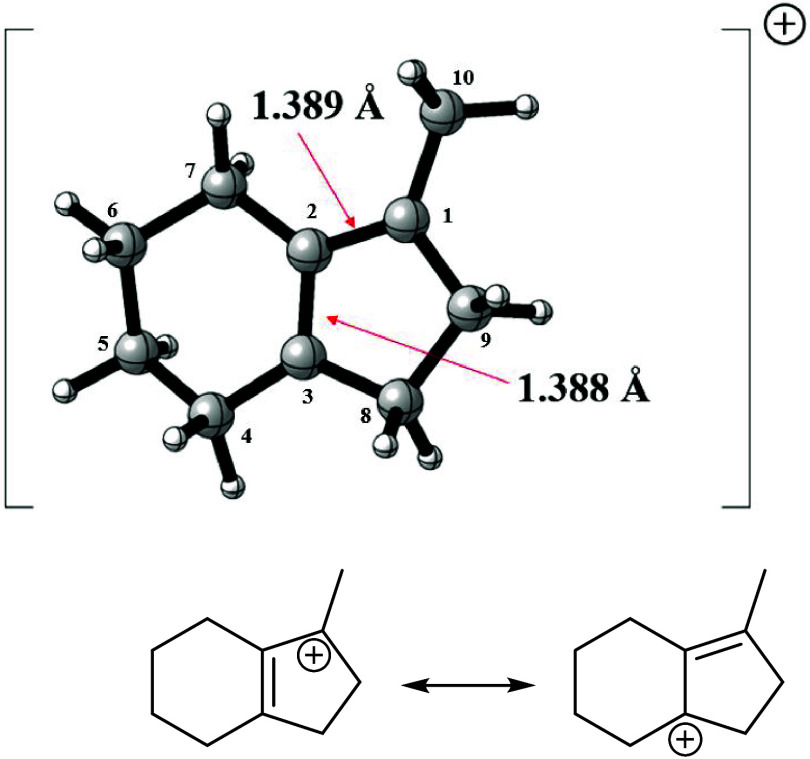
Structure of the 1-methyl-2,3,4,5,6,7-hexahydro-1*H*-indene-1-yl cation.

An interesting point about this isomer is that, upon analyzing the structure of carbocation, it becomes evident that there is no additional stabilization factor for the positive charge, apart from the fact that it is an allylic carbocation within a bicyclic system where the partial positive charge is always located at the tertiary position.

The ^13^C NMR chemical shifts of this indene cation in relation to TMS were predicted at GIAO/M062X/cc-pVTZ level as δ­(C_1_) = 291.2 ppm; δ­(C_2_) = 288.6 ppm; δ­(C_3_) = 180.1 ppm; δ­(C_4_) = 54.3 ppm; δ­(C_5_) = 51.6 ppm; δ­(C_6_) = 43.5 ppm; δ­(C_7_) = 29.0 ppm; δ­(C_8_) = 25.1 ppm; δ­(C_9_) = 22.4 ppm; δ­(C_10_) = 21.9 ppm. These results are in agreement with the literature data that show that the chemical shift for the carbon of allylic cation lies between 231.3 and 268.2 for the 1,3-substituted alkenyl carbocations studied by Olah and Spear,^24^ which was close to the theoretical value obtained for the cationic centers of the indene carbocation (291.2 and 288.6 ppm).

Interestingly, its carbon scaffold is related to many naphthenic compounds found in petroleum, which are characterized as saturated bicyclic hydrocarbons containing five or six-membered fused rings.^25^ These compounds can constitute up to 60% (m/m) of the extracted oil, and their formation in petroleum is recognized by the geochemical community as resulting from the biodegradation of petroleum or the thermal decomposition of heavier polycyclic natural products.^25^ However, since this carbocation is more stable than 1 and 2-adamantyl cation, the naphthenic and aromatic hydrocarbons formed from it may accumulate. This suggests that correlations between the composition of the naphthenic, diamantoids, and the biomarkers fractions in petroleum may exist, which may be of geochemical interest (Figure 12).

**Figure 12:**
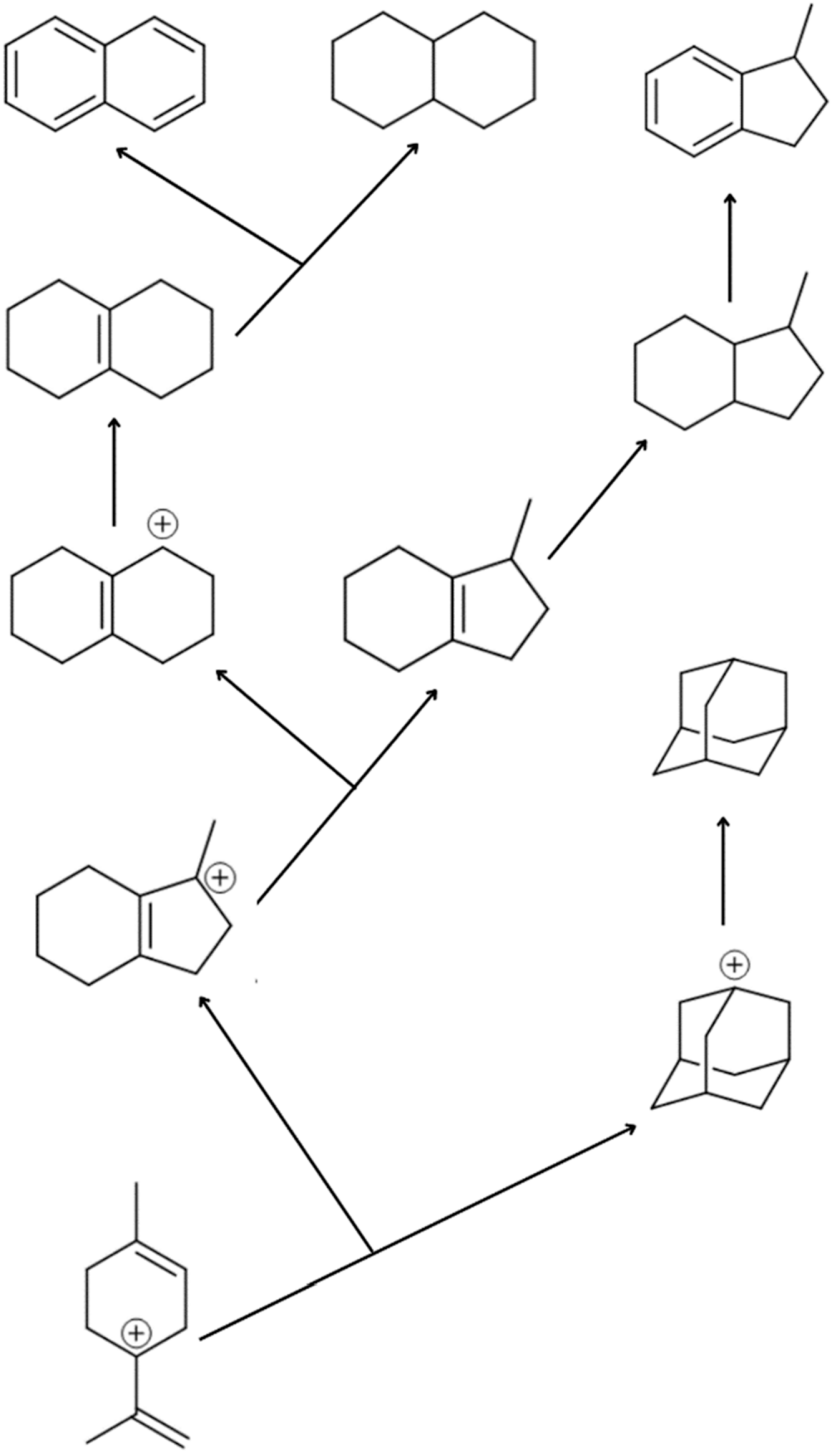
Evolution tree of C_10_ isomers in petroleum.

Thus, it is conceivable that carbocation rearrangement pathways involved in the formation of diamondoids and C_10_ naphthenic compounds in petroleum might be interconnected (Figure 11), allowing the evolutionary history of a given petroleum basin to be established. This can be interpreted from a new perspective, that the rearrangement of terpenoid carbocation isomers and related species.

## Conclusions

Based on the calculations performed at the M06–2X/cc-pVTZ level of theory for the known C_10_H_16_ isomers cataloged on the NIST platform, adamantane was identified as the most stable cataloged alkane, corroborating Olah’s ideas that this compound is the most stable isomer. Thus, it was possible to reaffirm the initial idea that this isomer would be a thermodynamic well for C_10_H_16_ isomers among the cataloged species. However, when searching for C_10_H_15_^+^ isomeric species with AUTOMATON, it was identified that other C_10_H_15_^+^ carbocations are more stable than the 1-adamantyl cation, which was previously recognized as a thermodynamic well along with its neutral isomer. The most stable species found is the 1-methyl-2,3,4,5,6,7-hexahydro-1*H*-indene-1-yl cation. The neutral compounds that may be formed from it, either by reduction to the hydrocarbon or aromatization, have the skeleton of naphthenic rings found in petroleum. Thus, a route for the rearrangement of carbocation was indicated, suggesting that petroleum naphthenes could also be formed by isomerization reactions. However, it is noteworthy to highlight that adamantane remains the most stable neutral isomer, indicating that its formation could also occur from C_10_H_15_^+^ carbocations isomerization reactions in petroleum through an as yet unelucidated mechanism.

## Supplementary Material



## Notes

Isomer structures, carbocation structures, and energies (PDF)
