# Supplementary material for: The Adamantaneland Revisited
Source: J Phys Chem A. 2025 Nov 4;129(45):10443–51. doi: 10.1021/acs.jpca.5c06236 (PMC12621239; doi:10.1021/acs.jpca.5c06236)
Supplement: Supplementary file 1 [file jp5c06236_si_001.pdf]

Supporting Information for

The Adamantaneland Revisited

Pedro H. Antunes Silva[a], Amir L. Perlin[a], Cleverson J. F. de Oliveira[b], Ricardo R. Oliveira[a], Pierre M. Esteves[a]*

[a] Instituto de Química, Universidade Federal do Rio de Janeiro, Av. Athos da Silveira Ramos, 149, CT A-622, Cid. Univ., Rio de Janeiro, RJ, 21941-909 – Brazil

[b] Centro de Pesquisa e Desenvolvimento Leopoldo Miguez de Mello, Cenpes (Petrobras), Rio de Janeiro, Brazil

*Corresponding author: pesteves@iq.ufrj.br

1. Isomers structures Engler and co. (1973)

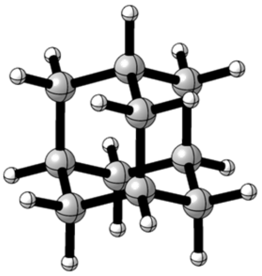
Adamantane

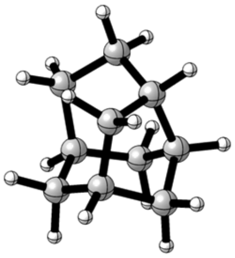
Alkane 3

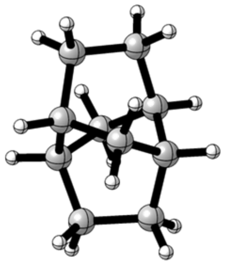
Alkane 4

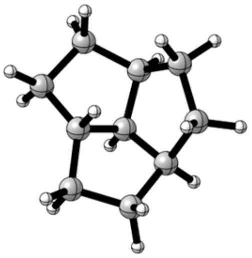
Alkane 5

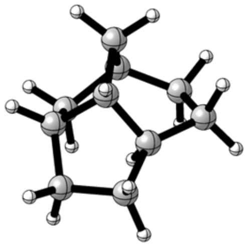
Alkane 6

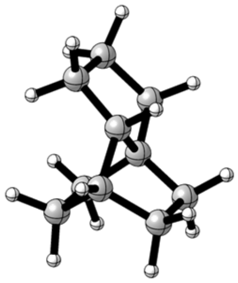
Alkane 7

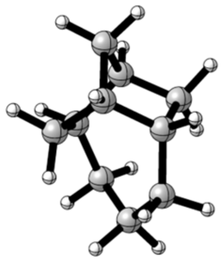
Alkane 9

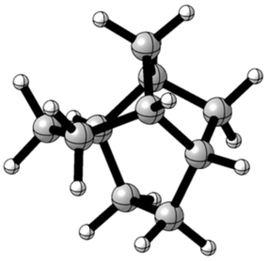
Alkane 10

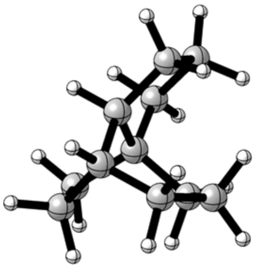
Alkane 12

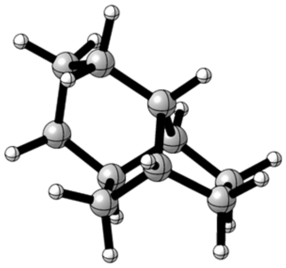
Alkane 13

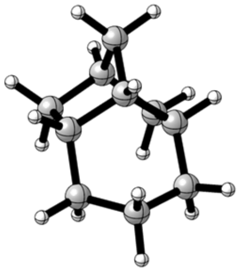
Alkane 14

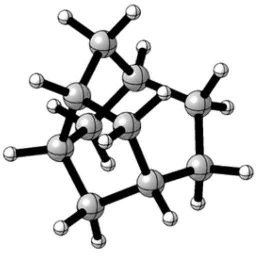
Alkane 20

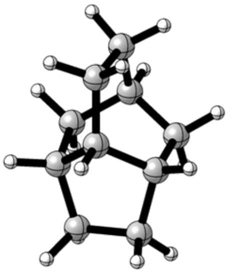
Alkane 21

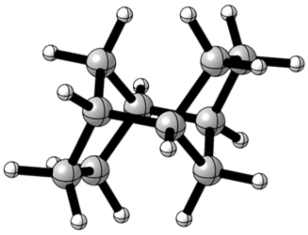
Anti 11

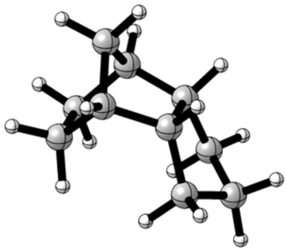
Endo 2

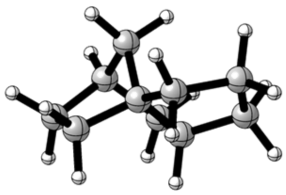
Endo 8

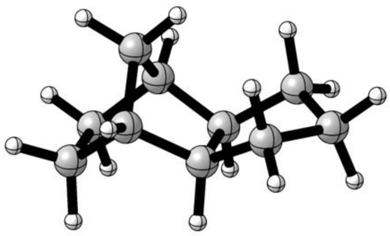
Exo 2

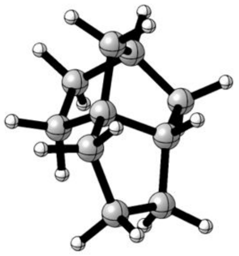
Exo 8

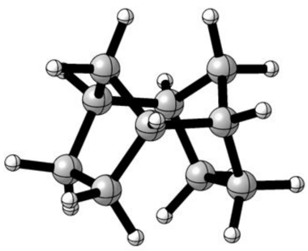
Syn 11

2. Carbocations Structures Engler and co. (1973)

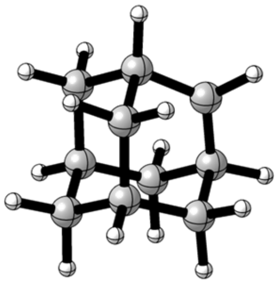
Cation 1

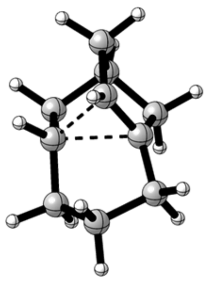
Cation 2

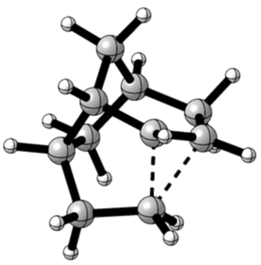
Cation 3

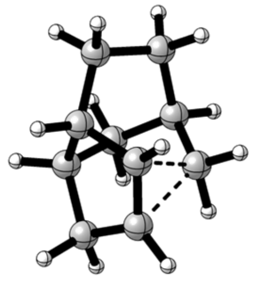
Cation 4

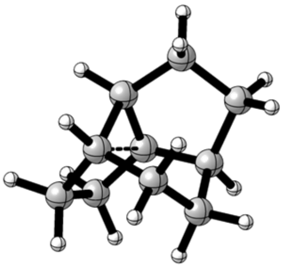
Cation 5

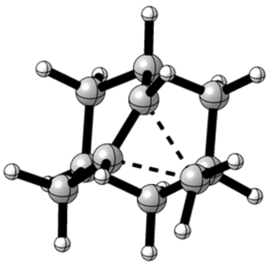
Cation 6

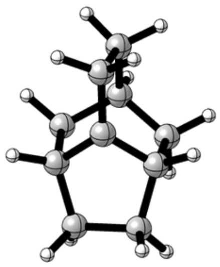
Cation 7

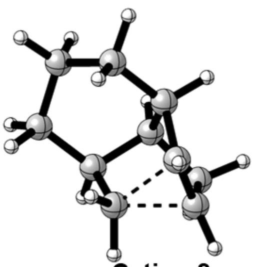
Cation 8

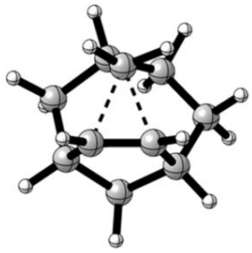

Cation 9

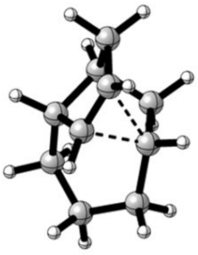

Cation 10

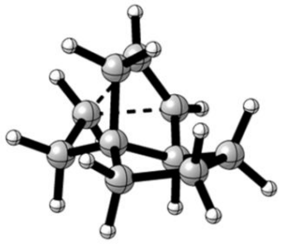

Cation 11

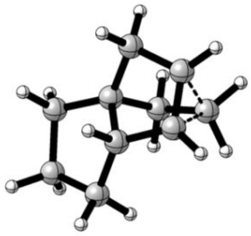

Cation exo8

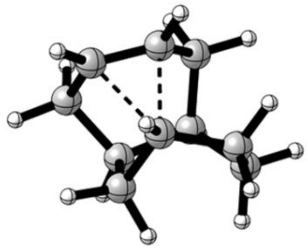

Cation 12

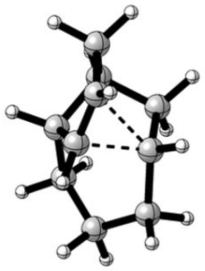

Cation 13

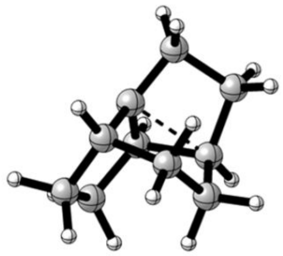

Cation 14

3. All $C_{10}H_{16}$ isomers below are catalogued in the NIST thermodynamics database

Isomer	Name	ΔG (Hartree)	ΔH (Hartree)	ΔS cal/mol.K
Adamantane	c10h16_20	-390.43130	-390.39260	81.46
2,5-Methano-1H-indene, octahydro	c10h16_47	-390.41483	-390.37570	82.37
Perhydrotriquinacene	c10h16_52	-390.41318	-390.37239	85.87
9,10-Octalin	c10h16_147	-390.41092	-390.36906	88.09
Exo-tricyclo[5.2.1.0(2.6)]decane	c10h16_113	-390.40858	-390.36804	85.31
bicyclo[4.4.0]-2-decene	c10h16_217	-390.40684	-390.36447	89.20
4,7-Methano-1H-indene, octahydro	c10h16_34	-390.40605	-390.36446	87.53
exo-Ttricyclo[5.2.1.0(2,6)]decane	c10h16_46	-390.40605	-390.36446	87.53
3,7-dimethylbicyclo[3.3.0]-2-octene	c10h16_192	-390.40352	-390.35828	95.21
Endo-tricyclo[5.2.1.0(2.6)]decane	c10h16_111	-390.40208	-390.36094	86.57
1,3-Cyclopentadiene, 1,2,3,4,5-pentamethyl	c10h16_94	-390.40187	-390.35140	106.24
1-methylbicyclo[4.3.0]-3-nonene	c10h16_200	-390.40175	-390.35864	90.72
1-Methyl-4-methylenebicyclo[3.2.1]oct-2-ene	c10h16_112	-390.40121	-390.35851	89.86
1,2,3,5,5 And 1,2,4,5,5-pentamethylcyclopentadiene mixture	c10h16_144	-390.39902	-390.34984	103.50
Cyclohexene, 1,5,5-trimethyl-3-methylene	c10h16_80	-390.39823	-390.35195	97.41
Spiro[4.5]dec-6-ene	c10h16_224	-390.39801	-390.35446	91.67
1,3-Cyclohexadiene, 1,3,5,5-tetramethyl	c10h16_55	-390.39750	-390.35042	99.09
γ-Pyronene	c10h16_60	-390.39750	-390.35042	99.09
Bicyclopentylidene	c10h16_131	-390.39725	-390.35294	93.25
4,4-dimethylbicyclo[3.2.1]-2-octene	c10h16_193	-390.39647	-390.35336	90.72
1(7)-Bicyclo[5.3.0]decane	c10h16_209	-390.39633	-390.35215	92.98

Silveterpinolene	c10h16_138	-390.39514	-390.34648	102.42
ε-Fenchene	c10h16_65	-390.39501	-390.35013	94.46
1-Cyclopentylcyclopentene	c10h16_90	-390.39490	-390.34868	97.28
1,2,3,4-tetramethyl-1,3-cyclohexadiene	c10h16_180	-390.39408	-390.34545	102.35
Bicyclo[2.2.2]oct-2-ene, 5-ethyl-, (1α,4α,5α)	c10h16_134	-390.39329	-390.34976	91.61
Bicyclopentyl-3-ene	c10h16_95	-390.39311	-390.36643	98.23
Spiro[4.4]nonane, 1-methylene	c10h16_106	-390.39305	-390.34840	93.98
4,7,7-trimethyltricyclo[2.2.1.02,6]heptane	c10h16_166	-390.39295	-390.34995	90.51
Tricyclo[2.2.1.0(2,6)]heptane, 1,7,7-trimethyl	c10h16_16	-390.39294	-390.34973	90.94
3-Cyclopentylcyclopentene	c10h16_91	-390.39243	-390.34605	97.63
Tricyclo[2.2.1.0(2,6)]heptane, 1,3,3-trimethyl	c10h16_41	-390.39239	-390.34863	91.46
1,3-Cyclohexadiene, 1-methyl-4-(1-methylethyl)	c10h16_10	-390.39222	-390.34494	99.49
1,5,5-Trimethyl-6-methylene-cyclohexene	c10h16_102	-390.39212	-390.34671	95.57
2,4,4-trimethyl-3-methylenecyclohexene	c10h16_179	-390.39212	-390.34671	95.57
Bicyclo[2.2.2]oct-2-ene, 5-ethyl-, (1α,4α,5β)	c10h16_137	-390.39209	-390.34860	91.53
Cyclohexene, 3-methyl-6-(1-methylethylidene)	c10h16_22	-390.39165	-390.34311	102.15
1,3-Cyclohexadiene, 1,2,6,6-tetramethyl	c10h16_50	-390.39158	-390.34459	98.90
1-methyl-4-propyl-1,3-cyclohexadiene	c10h16_188	-390.39138	-390.34340	100.99
Ƴ-terpineno	c10h16_5	-390.39118	-390.34298	101.45
Bicyclo[2.2.1]heptane, 2,2-dimethyl-5-methylene	c10h16_79	-390.38987	-390.34666	90.95
(+)-4-methyl-2-(1-methylethenyl)cyclohexene	c10h16_161	-390.38986	-390.34347	97.64
Bicyclo[2.2.1]heptane, 2,2-dimethyl-3-methylene-, (1R)	c10h16_45	-390.38975	-390.34638	91.27
Canphene	c10h16_8	-390.38974	-390.34638	91.26
Cyclohexene, 3-(2-methylpropylidene)-, (Z)	c10h16_237	-390.38968	-390.34055	103.41
1,3-diethyl-1,3-cyclohexadiene	c10h16_204	-390.38960	-390.34175	100.72
1,4-Cyclohexadiene, 3,3,6,6-tetramethyl	c10h16_97	-390.38956	-390.34334	97.26
Bicyclo[2.2.1]heptane, 7,7-dimethyl-2-methylene	c10h16_18	-390.38933	-390.34575	91.72
1,3-Cyclohexadiene, 1,5,5,6-tetramethyl	c10h16_88	-390.38905	-390.34298	96.96
Spiro[bicyclo[2.2.2]octane-2,1'-cyclopropane]	c10h16_135	-390.38858	-390.34646	88.65
Sylveterpinolene	c10h16_142	-390.38831	-390.33951	102.72
Cyclohexene, 1-methyl-4-(1-methylethylidene)	c10h16_7	-390.38826	-390.33941	102.80
α-Terpinolene	c10h16_39	-390.38826	-390.33941	102.80
(Z)-β-Terpinolene	c10h16_119	-390.38826	-390.33941	102.80
1-methyl-5-(1-methylethyl)-1,3-cyclohexadiene	c10h16_157	-390.38799	-390.34090	99.09
Cyclohexene, 4-methyl-1-(1-methylethenyl)	c10h16_35	-390.38766	-390.34083	98.56
(+)-4-methyl-1-(1-methylethenyl)cyclohexene	c10h16_160	-390.38766	-390.34083	98.56
(+)-(4S)-α-phellandrene	c10h16_43	-390.38714	-390.33978	99.68

2,5,5-Trimethylbicyclo[2.2.1]hept-2-ene	c10h16_92	-390.38710	-390.34310	92.62
γ-Fenchene	c10h16_66	-390.38710	-390.34310	92.62
δ-Terpinene	c10h16_48	-390.38709	-390.34006	98.99
δ-Terpinene	c10h16_124	-390.38709	-390.34006	98.99
1,5-diethyl-1,3-cyclohexadiene	c10h16_205	-390.38688	-390.33925	100.25
(.+-.)-3-methyl-1-(1-methylethenyl)cyclohexene	c10h16_155	-390.38687	-390.33996	98.73
(+)-3-methyl-1-(1-methylethenyl)cyclohexene	c10h16_163	-390.38687	-390.33996	98.73
α-Phellandrene	c10h16_11	-390.38681	-390.33965	99.25
(+)-α-phellandrene	c10h16_98	-390.38681	-390.33965	99.25
β-Phellandrene	c10h16_14	-390.38651	-390.33991	98.08
Cyclohexene, 1-methyl-5-(1-methylethenyl)-, (R)	c10h16_27	-390.38644	-390.33938	99.05
Cyclohexene, 1-methyl-5-(1-methylethenyl)	c10h16_175	-390.38644	-390.33938	99.05
(+)-1-methyl-3-(1-methylethenyl)cyclohexene	c10h16_159	-390.38637	-390.33882	100.06
m-Mentha-1,8-diene	c10h16_71	-390.38636	-390.33882	100.05
1-(2-methyl-1-propenyl)cyclohexene	c10h16_194	-390.38636	-390.33875	100.21
δ-phellandrene	c10h16_74	-390.38613	-390.33925	98.65
Limoleno	c10h16_58	-390.38608	-390.33927	98.54
limoleno	c10h16_1	-390.38585	-390.33861	99.42
D-Limonene	c10h16_30	-390.38585	-390.33861	99.42
Cyclohexene, 1-methyl-4-(1-methylethenyl)-, (S)	c10h16_29	-390.38584	-390.33861	99.41
trans-m-Mentha-2,8-diene	c10h16_127	-390.38576	-390.33894	98.56
Bicyclo[2.2.1]hept-2-ene, 2,7,7-trimethyl	c10h16_54	-390.38539	-390.34114	93.14
1,5,5-trimethylcyclohepta-1,3-diene	c10h16_125	-390.38536	-390.33941	96.70
Cyclohexene, 5-ethenyl-1,5-dimethyl	c10h16_73	-390.38521	-390.33922	96.79
Cyclohexene, 4-ethenyl-1,4-dimethyl	c10h16_107	-390.38508	-390.33902	96.94
δ-Fenchene	c10h16_67	-390.38500	-390.34155	91.46
1,5,5-trimethylbicyclo[2.2.1]-2-heptene	c10h16_173	-390.38500	-390.34154	91.47
β-fenchene	c10h16_40	-390.38500	-390.34154	91.47
2,6,6-trimethylcyclohepta-1,3-diene	c10h16_126	-390.38495	-390.33883	97.07
1(7),3,8-ortho -Menthatriene	c10h16_116	-390.38446	-390.33729	99.27
1-(2-methyl-3-propenyl)cyclohexene	c10h16_195	-390.38436	-390.33705	99.58
Cyclohexene, 4-methylene-1-(1-methylethyl)	c10h16_33	-390.38418	-390.33725	98.79
3,6-dimethyl-1,2-dimethylenecyclohexane	c10h16_172	-390.38383	-390.33810	96.26
Bicyclo[2.2.1]hept-2-ene, 1,7,7-trimethyl	c10h16_49	-390.38366	-390.34014	91.60
m-Mentha-4,8-diene, (1S,3S)-(+)	c10h16_69	-390.38357	-390.33632	99.46
1,3-Cyclopentadiene, 2-pentyl	c10h16_77	-390.38344	-390.33372	104.64
1,3-Cyclopentadiene, 1-pentyl	c10h16_78	-390.38331	-390.33337	105.10
3-ethenyl-1,5-dimethylcyclohexene	c10h16_197	-390.38328	-390.33635	98.77
3-methyl-5-(1-methylethenyl)cyclohexene	c10h16_176	-390.38304	-390.33617	98.63
3-methyl-4-(1-methylethenyl)cyclohexene	c10h16_177	-390.38297	-390.33524	100.47

(3S-trans)-3-methyl-6-(1-methylethenyl)cyclohexene	c10h16_151	-390.38276	-390.33548	99.49
Cyclohexene, 3-methyl-6-(1-methylethenyl)-, (3R-trans)	c10h16_42	-390.38275	-390.33548	99.49
1(7), 4(8)-P-menthadiene	c10h16_150	-390.38262	-390.33642	97.22
(+)-4-methyl-2-(2-propenyl)cyclohexene	c10h16_178	-390.38217	-390.33476	99.79
m-Mentha-1(7),8-diene	c10h16_76	-390.38211	-390.33575	97.57
Cyclohexane, 1-methylene-4-(1-methylethenyl)	c10h16_25	-390.38210	-390.33554	97.99
trans-4-methyl-5-(1-methylethenyl)cyclohexene	c10h16_156	-390.38206	-390.33547	98.05
Mentha-2,8-diene	c10h16_104	-390.38179	-390.33455	99.43
Cyclopentane, 2-methyl-1-methylene-3-(1-methylethenyl)	c10h16_222	-390.38165	-390.33313	102.11
2-Carene	c10h16_19	-390.38130	-390.33523	96.97
(+)-3-Carene	c10h16_32	-390.38130	-390.33523	96.96
(+)-2-Carene	c10h16_56	-390.37978	-390.33387	96.62
(1S-cis)-3,7,7-trimethylbicyclo[4.1.0]hept-2-ene	c10h16_100	-390.37978	-390.33387	96.62
cis-4-methyl-5-(1-methylethenyl)cyclohexene	c10h16_162	-390.37977	-390.33411	96.09
2-methyl-3-(1-methylethenyl)cyclohexene	c10h16_171	-390.37875	-390.33236	97.64
1,2,3-trimethyl-3,5-cycloheptadiene	c10h16_191	-390.37854	-390.33290	96.06
Cyclopentene, 3-isopropenyl-5,5-dimethyl	c10h16_96	-390.37816	-390.33069	99.92
1,3-Cyclopentadiene, 5-pentyl	c10h16_128	-390.37815	-390.32887	103.73
Bicyclo[3.1.0]hex-2-ene, 2-methyl-5-(1-methylethyl)	c10h16_12	-390.37802	-390.33131	98.31
Cyclobutadicyclopentene, decahydro	c10h16_240	-390.37754	-390.33587	87.70
Bicyclo[3.1.0]hex-2-ene, 4-methyl-1-(1-methylethyl)	c10h16_44	-390.37741	-390.33187	95.84
trans-Thujene	c10h16_86	-390.37689	-390.33011	98.45
cis-4-carene	c10h16_141	-390.37628	-390.33058	96.18
(+)-4-Carene	c10h16_28	-390.37611	-390.33076	95.44
Bicyclo[3.1.0]hexane, 4-methylene-1-(1-methylethyl)	c10h16_6	-390.37542	-390.32928	97.13
trans-Alloocimene	c10h16_31	-390.37519	-390.32223	111.48
2,4,6-Octatriene, 2,6-dimethyl	c10h16_17	-390.37519	-390.32223	111.48
2,4,6-Octatriene, 2,6-dimethyl-, (E,E)	c10h16_36	-390.37519	-390.32223	111.48
Tricyclo[3.3.2.02,8]decane	c10h16_221	-390.37513	-390.33407	86.41
2,4,6-Octatriene, 2,6-dimethyl-, (E,Z)	c10h16_53	-390.37507	-390.32173	112.27
endo-Ttricyclo[5.2.1.0(2,6)]decane	c10h16_114	-390.37433	-390.33372	85.49
cis-sabinene	c10h16_130	-390.37420	-390.32808	97.07
l-4,7,7-trimethylbicyclo[3.1.1]-2-heptene	c10h16_170	-390.37404	-390.32913	94.53
Cyclopentene, 1-(4-pentenyl)	c10h16_133	-390.37328	-390.32404	103.64
7,7-dimethyl-3-methylene-bicyclo[4.1.0]heptane	c10h16_121	-390.37322	-390.32815	94.85
Cyclopentane, 4-pentenylidene	c10h16_132	-390.37230	-390.32218	105.49
α-pineno	c10h16_2	-390.37215	-390.32773	93.50
1,5-Cyclooctadiene, 1,5-dimethyl	c10h16_70	-390.37156	-390.32518	97.61
(-)-α-thujene	c10h16_120	-390.37144	-390.32507	97.59
Spiro[2.4]heptane, 1,5-dimethyl-6-methylene	c10h16_226	-390.37095	-390.35368	99.48

(4Z,6z)-2,6-dimethyl-2,4,6-octatriene	c10h16_229	-390.37052	-390.31802	110.49
2,4,6-Octatriene, 3,4-dimethyl	c10h16_61	-390.37024	-390.31760	110.78
7,7-dimethyl-3-methylenebicyclo[4.1.0]heptane	c10h16_158	-390.37004	-390.32440	95.98
Dispiro[2.2.2.2]decane	c10h16_185	-390.36984	-390.32656	91.10
Dispiro[2.0.2.4]decane	c10h16_118	-390.36920	-390.32610	90.73
β-pineno	c10h16_4	-390.36874	-390.32480	92.48
Bicyclo[3.1.1]heptane, 6,6-dimethyl-2-methylene-, (1S)	c10h16_57	-390.36874	-390.32480	92.47
2,4-Dimethyl-2,4,6-octatriene	c10h16_117	-390.36857	-390.31634	109.91
(E,Z)-3,4-dimethyl-2,4,6-octatriene	c10h16_108	-390.36854	-390.31525	112.18
2,6-Dimethyl-octa-2,4,6-triene, cis	c10h16_59	-390.36828	-390.31435	113.51
1,4,6-Octatriene, 2,7-dimethyl	c10h16_123	-390.36769	-390.31452	111.92
(+)-γ-Pinene	c10h16_75	-390.36754	-390.32351	92.67
Cyclopentane, 3-methyl-1-(2-methylpropenylidene)	c10h16_122	-390.36753	-390.31785	104.57
trans,trans-1,6-Cyclodecadiene	c10h16_101	-390.36613	-390.32090	95.19
1,5-Cyclodecadiene, (E,Z)	c10h16_99	-390.36562	-390.32081	94.32
dl-4,7,7-trimethylbicyclo[3.1.1]-2-heptene	c10h16_165	-390.36439	-390.32055	92.26
2-Methyl, 6-methylene 2,4-octadiene	c10h16_233	-390.36390	-390.31173	109.79
2,3,3-trimethyltricyclo[2.2.1.02,6]heptane	c10h16_167	-390.36266	-390.31888	92.15
(3E,5z)-2,6-dimethyl-1,3,5-octatriene	c10h16_228	-390.36257	-390.31090	108.74
trans-β-Ocimene	c10h16_9	-390.36255	-390.31046	109.57
1,6-Cyclodecadiene	c10h16_140	-390.36253	-390.31762	94.52
β-Ocimene	c10h16_24	-390.36253	-390.31046	109.57
2,3,4,5-tetramethyl-1,3,5-hexatriene	c10h16_189	-390.36235	-390.31074	108.63
2,6-Dimethyl 1,3,5-octatriene (trans)	c10h16_231	-390.36230	-390.31101	107.95
1,5-Cyclooctadiene, 3,4-dimethyl	c10h16_203	-390.36213	-390.31605	96.99
1,1-dimethyl-2-methylene-3-(1-methylethylidene)cyclobutane	c10h16_146	-390.36167	-390.31160	105.39
1,3,6-Octatriene, 3,7-dimethyl-, (Z)	c10h16_13	-390.36150	-390.30957	109.30
Octane, 2,6-dimethyl-, hexadehydro deriv	c10h16_51	-390.36084	-390.30928	108.51
2,6-Dimethyl 1,4,6(7)-octatriene (cis-6(7))	c10h16_227	-390.36083	-390.31027	106.42
1,3,7-Octatriene, 3,7-dimethyl	c10h16_21	-390.36079	-390.30857	109.92
Cyclohexane, 1-butenylidene	c10h16_235	-390.36074	-390.31874	101.66
1,3,6-Heptatriene, 2,5,6-trimethyl	c10h16_234	-390.36049	-390.30988	106.51
1,5-Heptadiene, 2,5-dimethyl-3-methylene	c10h16_68	-390.36012	-390.30788	109.96
1,3,6-Heptatriene, 2,5,5-trimethyl	c10h16_38	-390.35984	-390.30973	105.46
(3-butynyl)cyclohexane	c10h16_218	-390.35934	-390.31209	99.44
1,2-bis(1-methylethylidene)cyclobutane	c10h16_181	-390.35865	-390.30932	103.83
Isomyocorene	c10h16_190	-390.35757	-390.30650	107.48
cis,cis-1,6-Cyclodecadiene	c10h16_72	-390.35730	-390.31300	93.24
cis,cis-1,6-cyclodecadiene	c10h16_212	-390.35730	-390.31300	93.23
2-Hexen-4-yne, 2-methyl-3-(1-methylethyl)	c10h16_202	-390.35664	-390.30110	116.89
β-mirceno	c10h16_3	-390.35638	-390.30387	110.51

Santolina triene	c10h16_23	-390.35625	-390.30432	109.30
Tricyclo[7.1.0.0(4,6)]decane	c10h16_89	-390.35603	-390.31367	89.15
5,5-Dimethyl-1-vinylbicyclo[2.1.1]hexane	c10h16_103	-390.35453	-390.30923	95.36
1,1,2,2-tetramethyl-3,4-dimethylenecyclobutane	c10h16_154	-390.35436	-390.30723	99.18
(E)-4,6-Dimethyl-1,3,7-octatriene	c10h16_115	-390.35399	-390.30180	109.84
1,7-Octadiene, 2-methyl-6-methylene	c10h16_62	-390.35354	-390.30166	109.19
Cyclobutane, 1,2-bis(1-methylethenyl)-, trans	c10h16_93	-390.35311	-390.30451	102.29
Tricyclo[6.2.0.03,6]decane	c10h16_241	-390.34996	-390.30833	87.63
cyclodecyne	c10h16_232	-390.34935	-390.30341	96.68
4-Decen-6-yne, (Z)	c10h16_109	-390.34769	-390.29102	119.28
anti-Tricyclo[7.1.0.0]decane	c10h16_136	-390.34601	-390.30263	91.29
2-Methyl-1-nonene-3-yne	c10h16_145	-390.34558	-390.29069	115.52
trans-1,2-Diethenyl-1,2-dimethylcyclobutane	c10h16_143	-390.34556	-390.29890	98.20
(4Z,6e)-2,6-dimethyl-2,4,6-octatriene	c10h16_230	-390.34350	-390.29137	109.71
Tricyclo[4.2.2.0^2,5]decane	c10h16_214	-390.34331	-390.30334	84.11
4-Decen-6-yne, (E)	c10h16_110	-390.34299	-390.29132	108.75
2-methyl-nonen-4-yne	c10h16_223	-390.34109	-390.28984	107.88
1-Decen-3-yne	c10h16_139	-390.34038	-390.28555	115.39
1,2-diethenyl-3,4-dimethylcyclobutane	c10h16_183	-390.33940	-390.29142	100.98
3-Carene	c10h16_15	-390.33935	-390.29379	95.89
1-Methyl-trans-2-(cis-2,3-methylene)-4-pentenyl-cyclopropane	c10h16_82	-390.33891	-390.28936	104.28
Cyclopropane,1,1'-(2-methyl-1-propenylidene)bis	c10h16_153	-390.33830	-390.29004	101.58
Cyclobutane,2-ethynyl-1,1,3,3-tetramethyl	c10h16_201	-390.33820	-390.29024	100.93
(cis-1,2-Methylene)-trans-4-hexenyl-cyclopropane	c10h16_85	-390.33783	-390.28798	104.93
1-Methyl-trans-2-(trans-2,3-methylene)-4-pentenyl-cyclopropane	c10h16_81	-390.33779	-390.28847	103.79
(trans-1,2-Methylene)-trans-4-hexenyl-cyclopropane	c10h16_83	-390.33746	-390.28694	106.33
3-Decen-1-yne, (Z)	c10h16_184	-390.33682	-390.28328	112.68
(trans-4,5-Methylene)-trans-1-hexenyl-cyclopropane	c10h16_84	-390.33665	-390.28728	103.91
3-Decen-1-yne, (E)	c10h16_187	-390.33586	-390.28276	111.75
Cyclopropane, trimethyl(2-methyl-1-propenylidene)	c10h16_152	-390.33081	-390.27859	109.92
cis-1-Methyl-1,2-dicyclopropylcyclopropane	c10h16_64	-390.32997	-390.28238	100.15
trans-1-Methyl-1,2-dicyclopropylcyclopropane	c10h16_63	-390.32991	-390.28208	100.68
bicyclo[4.4.0]-1-decene	c10h16_216	-390.31472	-390.27222	89.45
2,8-P-menthadiene	c10h16_239	-390.29758	-390.25127	97.45
(1S)-2,6,6-Trimethylbicyclo[3.1.1]hept-2-ene	c10h16_26	-390.27829	-390.23096	99.61
α-Pinene	c10h16_225	-390.27829	-390.23096	99.60
Bicyclo[4.2.1]nonane,9-methylene	c10h16_199	-390.19457	-390.15251	88.53
Cyclobutane, 1,2-dipropenyl	c10h16_164	-389.68239	-389.63080	108.58
d-4,7,7-trimethylbicyclo[3.1.1]-2-heptene	c10h16_169	-351.09600	-351.05298	90.55

(1R)-2,6,6-Trimethylbicyclo[3.1.1]hept-2-ene	c10h16_37	-351.08867	-351.04779	86.03
Alpha cis ocimene	c10h16_238	-351.07865	-351.03103	100.23
